# Profiling sex-biased gene expression during parthenogenetic reproduction in *Daphnia pulex*

**DOI:** 10.1186/1471-2164-8-464

**Published:** 2007-12-18

**Authors:** Brian D Eads, John K Colbourne, Elizabeth Bohuski, Justen Andrews

**Affiliations:** 1 The Center for Genomics and Bioinformatics and Department of Biology, Indiana University Bloomington, Indiana 47405, USA

## Abstract

**Background:**

Sexual reproduction is a core biological function that is conserved throughout eukaryotic evolution, yet breeding systems are extremely variable. Genome-wide comparative studies can be effectively used to identify genes and regulatory patterns that are constrained to preserve core functions from those that may help to account for the diversity of animal reproductive strategies. We use a custom microarray to investigate gene expression in males and two reproductive stages of females in the crustacean *Daphnia pulex*. Most *Daphnia *species reproduce by cyclical parthenogenesis, alternating between sexual and clonal reproduction. Both sex determination and the switch in their mode of reproduction is environmentally induced, making *Daphnia *an interesting comparative system for the study of sex-biased and reproductive genes.

**Results:**

Patterns of gene expression in females and males reveal that 50% of assayed transcripts show some degree of sex-bias. Female-biased transcription is enriched for translation, metabolic and regulatory genes associated with development. Male-biased expression is enriched for cuticle and protease function. Comparison with well studied arthropods such as *Drosophila melanogaster *and *Anopheles gambiae *suggests that female-biased patterns tend to be conserved, whereas male-biased genes are evolving faster in *D. pulex*. These findings are based on the proportion of female-biased, male-biased, and unbiased genes that share sequence similarity with proteins in other animal genomes.

**Conclusion:**

Some transcriptional differences between males and females appear to be conserved across Arthropoda, including the rapid evolution of male-biased genes which is observed in insects and now in a crustacean. Yet, novel patterns of male-biased gene expression are also uncovered. This study is an important first step towards a detailed understanding of the genetic basis and evolution of parthenogenesis, environmental sex determination, and adaptation to aquatic environments.

## Background

Differences between males and females have fascinated biologists and stimulated research for many years, yet detailed molecular explorations of sexually dimorphic phenotypes are available for only a few organisms (cf. [1-5]). Among the insects, sex-biased gene expression is particularly well studied in *Drosophila *[6-13]. This research reveals that a large proportion of the transcriptome is sex-biased [2,10,12], and that most of these differences can be ascribed to divergence in the germ line [6,11]. Furthermore, sex is a much stronger determinant of transcript levels than an animal's ontogeny [7], age, or genotype [10]. These studies also find that, overall, male-biased genes are evolving more rapidly than female-biased genes [9,13,14], providing evidence that males experience stronger positive selection than females [8,12]. In addition, male genes are under-represented on the X chromosome of *D. melanogaster *[15]. To date, the generality of these findings for arthropods is not clear. Recent studies of *Anopheles gambiae *[3,16] report that a large proportion of gene expression is sex-biased and shows similar functional patterns to the female-biased gene set in flies. For example, females of both insects tend to express genes involved in ribosome function, translation initiation, and DNA replication at a higher level than males [16]. However, genes expressed at a two-fold or greater difference in *Drosophila *tend to be male-biased [11]. In contrast, 71% of highly biased genes are enriched in *Anopheles *females [16]. Further comparative experimental work is required to better understand the evolution of sex-biased gene expression.

Genome sequencing projects serve to underscore both the unity of eukaryotic organization as well as its amazing diversity. Functional characterization of genomes demonstrates that significant portions of gene inventories are preserved, which accounts for many of the core biological attributes defining large classes of organisms. In practice, comparative data from closely related species are a useful resource to ascribe tentative functions to conserved genomic elements, as studies of insects have begun to demonstrate [17,18]. Yet, investigations that include species from the other major lineages of arthropods (chelicerates and crustaceans) are equally important to discern insect-specific and arthropod-specific differences, and to infer ancestral versus derived conditions. Crustaceans are an amazingly varied group encompassing over 38,000 known species [19], and constitute a significant portion of animal communities in aquatic environments. Amongst crustaceans, the "water flea" *Daphnia *is arguably the best studied and has traditionally been an important model for ecology, population genetics, evolutionary biology, and toxicology [20]. Although these studies have focused on a variety of aspects of *Daphnia *biology, including phylogeography, functional morphology, physiology and life history evolution, genomic investigations have begun only recently [21]. A few large-scale studies of gene expression patterns in *Daphnia *have been published [22,23], but molecular studies have mostly been confined to a limited number of allozymes, sequence-tagged sites (STS), or genes [24,25]. Interest in crustacean biology *per se*, as well as their phylogenetic position as an outgroup to other well studied arthropods (*Drosophila, Apis, Anopheles*), emphasize the relevance of *Daphnia *in comparative genomic studies.

Several features of *Daphnia *biology make them an interesting subject of investigation in comparative functional genomics. Females typically reproduce by apomixis (parthenogenesis), depositing multiple embryos into a dorsal brood pouch that develop and hatch within about 48 hours [26]. Adverse environmental conditions such as crowding, lowered temperature or food scarcity can induce either male production (males are genetic copies of their mothers), or haploid gametes that are fertilized and overwinter in a state of diapause. Male production is known to be under the control of endocrine factors during oocyte maturation, and can be induced by juvenile hormone analogs such as the insecticides pyriproxyfen and fenoxycarb [27]. The ability to produce either males or haploid (sexual) eggs has been lost independently in multiple lineages of *Daphnia*, suggesting a simple genetic mechanism. In *D. pulex*, current data support the existence of a dominant allele conferring obligate asexuality in females but not in males [28], which has been spreading into North America from the northeast to the midwest [29]. As a step towards understanding these processes at a molecular level, here we specifically address the question of conservation of gene expression patterns and protein evolution in sex-biased genes.

We report here on gene expression profiles of *D. pulex*, an emerging model for functional genomics, by measuring transcription levels in males and females at two stages of development using a custom cDNA microarray. We compare the gene expression profiles of *D. melanogaster *to those of *D. pulex *to discover conserved elements of sex-specific transcriptional regulation. Analysis of the patterns associated with sex-bias also reveals a conserved trend in male-biased genes to be evolving faster than female-biased.

## Results

### Profiling sex- and parthenogenesis- biased gene expression

Our study represents the first description of global patterns of sex-biased transcription in a crustacean species, and we analyzed our results using two major approaches. First, we compared our male and female profiles to each other and to published studies of other arthropods using annotated gene ontology functions and processes. Secondly, we used an evolutionary approach to assess patterns of sequence conservation and sex bias. Furthermore we tested mature females carrying their first brood as well as juvenile females, whose ovaries are beginning the reproductive cycle, in order to examine embryogenesis as well as oogenesis.

**Figure 1:**
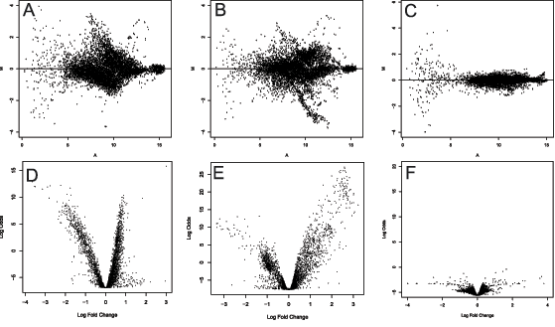
**Ratio vs. intensity and log odds vs. fold change plots**. (A, D) Males vs. juvenile females; (B, E) males vs. mature females; (C, F) self vs. self hybridizations. Ratio-intensity plots (A-C) depict values for a representative experimental replicate, while log odds-fold change plots (D-F) are based on four replicate hybridizations.

Examining patterns of gene expression in females (either juvenile or mature) compared to males reveals that the majority of array elements show some degree of sex-biased gene expression (Fig. 1). We tested mature females carrying their first brood as well as juvenile females, whose ovaries are beginning the reproductive cycle, to examine embryogenesis as well as oogenesis. Analysis of microarray data indicated that the arrays and the hybridizations are of high quality, characterized by excellent reproducibility between experiments, good dynamic range and low noise. After normalization, the intraslide correlation between duplicate spots was greater than 0.99, and the interslide correlation was greater than 0.94. The coefficient of variation (CV) for spots combined across arrays ranged from 0.003–0.50, with a mean (± S.D.) of 0.04 ± 0.047 (n = 3,673), indicating a negligible effect of spot intensity on variation. The high reproducibility of the experiments allowed the detection of statistically significant differences of approximately 1.25-fold (Fig. 1, Additional file 1).

We used the number of array elements and assembled sequences, fully described in reference [30], to estimate the number of unique genes differentially expressed in each comparison (Table 1). When data from the male-mature female and male-juvenile female experiments were pooled, a total of 81% (1,270) of genes shows statistically significant sex-biased gene expression with an estimated false discovery rate of 5% (Table 1). With a more conservative threshold of both at least a two-fold difference and statistical significance, a total of 45% (698) of genes, corresponding to 1,355 array elements, showed sex-biased gene expression (Table 1). This overall pattern is consistent with previous studies of other arthropods [2,3,12]. Additionally, there were almost three times as many male enriched genes compared to female enriched genes (957 versus 313, Table 1). However, at a two-fold or greater expression difference the proportion of male-biased transcription declined substantially (431 male biased and 267 female biased).

**Figure 2:**
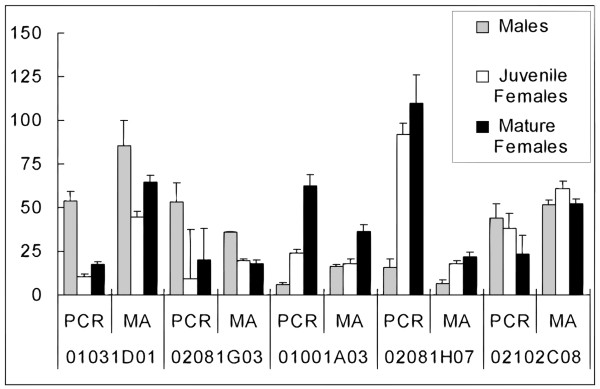
**Comparison of microarray (MA) and quantitative RT-PCR (PCR) results**. For MA results, normalized ratio and intensity values were used to back-calculate red and green fluorescence intensity for each experimental comparison and are reported as mean ± one standard error. For PCR results, standard curves were used to generate estimates of starting amounts for each reaction on a relative scale. Biological and technical replicates were combined for each group to calculate mean ± one standard error.

In addition, we used real-time reverse transcription PCR (RT-PCR) to validate the microarray results for five selected genes, and found concordance for the direction of differential expression in each case, including a non-differentially expressed transcript (Fig. 2). For example, for our element 01031D01, expression levels measured by microarray were 85 for males, 45 for juvenile females, and 65 for pregnant females (relative fluorescence intensity). When measured by RT-PCR, this gene was expressed at 54, 10, and 18 relative fluorescence units for males, juvenile females, and pregnant females, respectively. Our results thus show a compression of ratios in microarray results compared to RT-PCR, similar to other published comparisons [31,32].

**Table 1:** Differentially expressed genes in male and female *D. pulex*

	Mature Enriched vs. Male	Mature Depleted vs. Male	Juvenile Enriched vs. Male	Juvenile Depleted vs. Male	Self vs. Self	Male enriched vs. Female	Female Enriched vs. Male	Mature enriched vs. Juvenile	Juvenile enriched vs. Mature
Total Significant*	775	1183	732	1151	0	1445	757	591	773
Sequenced	465	554	391	514	-	590	432	259	629
Assembled	201	321	191	349	-	391	179	159	196
Estimated Non-redundant Genes^†^	335 (21%)	685 (44%)	357 (23%)	781 (50%)	-	957 (61%)	313 (20%)	363 (23%)	241 (15%)
Two-fold Difference	247	431	362	13	0	738	617	23	388
Sequenced	198	234	136	9	-	361	385	11	249
Assembled	91	128	136	7	-	211	167	8	122
Estimated Non-redundant Genes^†^	113 (7.2%)	235 (15%)	216 (14%)	10 (0.6%)	-	431 (28%)	267 (17%)	19 (1.2%)	190 (12%)

All differentially expressed array elements and assembled genes in male-female comparisons and at a two-fold difference. Comparisons include male vs. mature female, male vs. juvenile female, males vs. females (combined), mature vs. juvenile females, and self vs. self. *Total numbers of significant elements at 5% false discovery rate. † For each category (column), estimated non-redundant genes are calculated as proportion: assembled sequences/sequenced elements multiplied by total number significant. Percentages are calculated relative to the estimate of 1,560 unique genes on the array.

A closer examination revealed important differences between the patterns of sex-biased gene expression in juvenile and mature females. At the gross level, both males and mature females displayed a paucity of highly differentially expressed genes relative to juvenile females. Mature females expressed 21% of genes and juvenile females expressed 23% of genes at a two-fold or lower level relative to males (Table 1). By comparison, at a two-fold or higher level, males expressed 15% of genes more highly than mature females but only 0.6% of genes more highly than juvenile females. It is important to note that the arrayed cDNA libraries were made from mixed cultures comprised predominantly of females, and consequently are expected to under-represent male-specific and strongly male-biased genes. As males are a common reference, this indicates that a significant number of genes were also differentially expressed in juvenile females relative to mature females.

**Figure 3:**
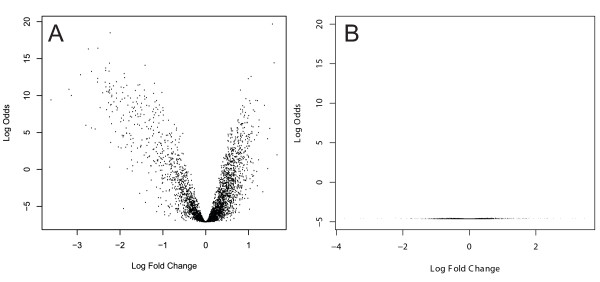
**Log-odds vs. fold changes males and females**. Using a linear model, log-odds and log fold changes were calculated for pregnant females vs. juvenile females (A) and male vs. male comparisons (B) and plotted based on four experimental replicates.

To verify, we used a linear model to quantitatively analyze differences between pregnant and juvenile females. The method implemented in limma [33] creates normalized intensity values for each target, which allowed comparisons to be made between groups, even when they were not directly compared. In effect, males served as a reference in this analysis. Using this approach, and by comparing male replicates to each other as a control, we identified genes differentially expressed between juvenile and pregnant females (Fig. 3A). As expected, the male-male comparison showed only two differentially expressed elements (Fig. 3B), while there were 604 differentially expressed genes between females, with 363 more highly expressed in mature females and 241 more highly expressed in juvenile females. At a two-fold difference threshold, mature females exhibited a substantially lower proportion (1.2%, 19 genes) compared to juveniles (12%, 190 genes, Table 1).

**Figure 4:**
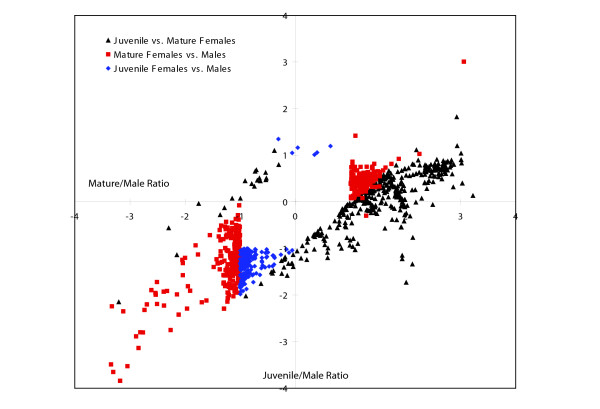
**Scatterplot of highly differentially expressed genes**. All significantly differentially expressed genes at two-fold change or above between males and mature females (X-axis) and between males and juvenile females (Y-axis) are depicted with male vs. mature as red squares; male vs. juvenile as blue diamonds; and mature vs. juvenile females as black triangles.

To examine the distribution of differentially expressed genes in the two comparisons of males and females, we compared transcripts differing by two-fold or greater between males and juveniles on the Y-axis and males and mature females on the X-axis of a scatterplot (Fig. 4). In this plot, negative ratios are female-enriched and positive ratios are male-enriched, so the lower left quadrant contains female-enriched and the upper right male-enriched genes. There are three important points illustrated by Figure 4. First, it is clear how few genes are relatively depleted in juvenile females, especially those enriched in males. This is illustrated by the small number of blue diamonds above the X-axis compared to below. Second, differences between juvenile and mature females, which are depicted as black triangles above and below the line of unity (Fig. 4), are quite substantial but also asymmetric. Juvenile-enriched genes are predominantly in the quadrants to the right of the Y-axis and are much more numerous than mature-enriched transcripts (Table1). Third, differences between mature females and males display a more even distribution, as shown by the clusters of red squares in Figure 4. There are more genes that are highly female-enriched, consistent with a smaller number of male-specific or highly male enriched transcripts present in the arrayed cDNA libraries.

### Functional categories of differentially expressed genes

The role of homology in inferring putative function for unknown gene sequences is well established and is the basis for annotation using a structured vocabulary such as the popular gene ontology, or GO [34,35]. One reason for this popularity is the ability to statistically test associations between GO annotations and gene expression measurements, although unfortunately no ontological or methodological approach can be considered optimal [36-38]. To investigate whether differentially expressed genes have a non-random representation of functional categories with respect to each other, we analyzed the occurrences of GO terms in each of the classes of differentially expressed genes (using assembled sequences only, so redundant sequences were not counted twice). We extracted the GO terms of the best Blast match in the GenBank NR database, and analyzed the relative frequency of differentially expressed genes to the reference set of all data using Fisher's exact test implemented in Blast2GO [39].

**Figure 5:**
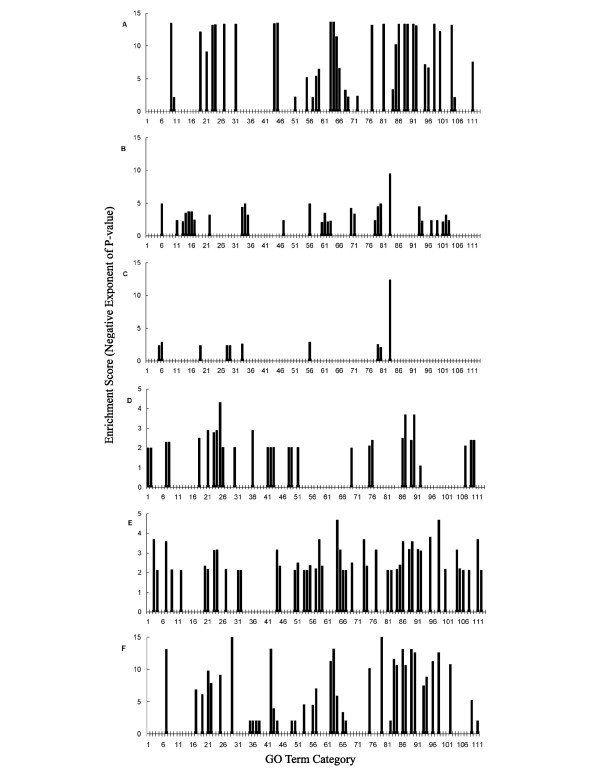
**Analysis of Gene Ontology (GO) terms**. Genes were categorized as significantly differentially expressed in a two-way comparison with false discovery rate correction and p < 0.05, and information about associated GO terms was extracted using Blast2GO [39]. Fisher's exact test was used to calculate the probability that the observed distribution of terms in the test set was significantly different (p-value < 0.05) from the distribution of all assembled sequences for the following comparisons: A) females vs. males; B) males vs. females; C) juvenile females vs. mature females; D) mature females vs. juvenile females; E) mature females vs. males; F) *Daphnia *and *Drosophila *conserved sex-biased genes. GO terms for each column are: 1, chromatin; 2, nucleosome; 3, nucleotide binding; 4, microtubule cytoskeleton organization and biogenesis; 5, lytic vacuole; 6, pattern binding; 7, nucleic acid binding; 8, DNA binding; 9, structural constituent of ribosome; 10, translation elongation factor activity; 11, catalytic activity; 12, GTPase activity; 13, endopeptidase activity; 14, serine-type endopeptidase activity; 15, chymotrypsin activity; 16, trypsin activity; 17, hydrolase activity, hydrolyzing O-glycosyl compounds; 18, chitinase activity; 19, structural molecule activity; 20, GTP binding; 21, cellular component; 22, extracellular region; 23, intracellular; 24, cell; 25, nucleus; 26, chromosome; 27, cytoplasm; 28, lysosome; 29, vacuole; 30, endoplasmic reticulum; 31, ribosome; 32, microtubule; 33, carbohydrate metabolism; 34, polysaccharide metabolism; 35, chitin metabolism; 36, nucleobase, nucleoside, nucleotide and nucleic acid metabolism; 37, DNA packaging; 38, establishment and/or maintenance of chromatin architecture; 39, chromatin assembly or disassembly; 40, nucleosome assembly; 41, transcription; 42, transcription, DNA-dependent; 43, regulation of transcription, DNA-dependent; 44, protein biosynthesis; 45, translational elongation; 46, protein complex assembly; 47, proteolysis and peptidolysis; 48, amino acid and derivative metabolism; 49, amino acid metabolism; 50, aromatic compound metabolism; 51, organelle organization and biogenesis; 52, chromosome organization and biogenesis (sensu Eukaryota); 53, cytoskeleton organization and biogenesis; 54, microtubule-based process; 55, physiological process; 56, chitin binding; 57, translation factor activity, nucleic acid binding; 58, biological_process; 59, metabolism; 60, peptidase activity; 61, serine-type peptidase activity; 62, catabolism; 63, macromolecule catabolism; 64, biosynthesis; 65, macromolecule biosynthesis; 66, cellular process; 67, microtubule cytoskeleton; 68, small ribosomal subunit; 69, cell organization and biogenesis; 70, hydrolase activity; 71, hydrolase activity, acting on glycosyl bonds; 72, isomerase activity; 73, purine nucleotide binding; 74, guanyl nucleotide binding; 75, regulation of nucleobase, nucleoside, nucleotide and nucleic acid metabolism; 76, regulation of metabolism; 77, protein metabolism; 78, protein catabolism; 79, carbohydrate binding; 80, polysaccharide binding; 81, ribonucleoprotein complex; 82, microtubule polymerization or depolymerization; 83, structural constituent of cuticle; 84, translation; 85, macromolecule metabolism; 86, organelle; 87, membrane-bound organelle; 88, non-membrane-bound organelle; 89, intracellular organelle; 90, intracellular membrane-bound organelle; 91, intracellular non-membrane-bound organelle; 92, protein complex; 93, biopolymer metabolism; 94, biopolymer catabolism; 95, cellular metabolism; 96, primary metabolism; 97, cellular catabolism; 98, cellular biosynthesis; 99, cellular protein catabolism; 100, cellular macromolecule metabolism; 101, cellular carbohydrate metabolism; 102, cellular polysaccharide metabolism; 103, cellular macromolecule catabolism; 104, cellular protein metabolism; 105, translation regulator activity; 106, tubulin; 107, regulation of transcription; 108, microtubule polymerization; 109, regulation of biological process; 110, regulation of physiological process; 111, cellular physiological process; 112, protein polymerization; 113, chromosome organization and biogenesis.

Female-biased genes are significantly enriched for a variety of GO categories involving protein metabolism, especially synthetic pathways relating to ribosome function and translation elongation (Fig. 5A). Other enriched categories identified macromolecule metabolism and primary metabolism, including both anabolic and catabolic functions. Organelle function and synthesis are also represented by multiple categories (Fig. 5A). Male-enriched *D. pulex *genes on our array correspond to 30 over-represented GO categories, compared to 38 for females (Fig 5B). Male-enriched GO terms generally related to either cuticle metabolism or serine peptidases, which are thought to have roles in cellular signalling and defence as well as digestion. Cuticle metabolism includes functional categories such as carbohydrate binding, polysaccharide metabolism, and structural constituent of cuticle functions (Fig. 5B).

Analysis of GO term enrichment in juvenile compared to mature females reveals differences that can be ascribed to embryogenesis. Specifically, while juvenile females show a paucity of categories enriched vs. mature females, mature females are enriched for a number of GO terms (5C, 5D). Over-represented GO terms in juvenile females include lytic vacuole, lysosome, vacuole, structural molecule activity, carbohydrate metabolism, and structural constituent of cuticle (Fig. 5C). Both males and juvenile females, which are approximately half the size of mature females, are significantly enriched for exoskeletal-associated gene function. This enrichment may be a simple consequence of larger surface area/volume ratios, which presumably require a higher concentration of cuticular proteins per unit mass. For mature females, enriched GO categories include those related to DNA packaging as well as transcription (Fig. 5D). Other GO terms are associated with amino acid metabolism and organelle function. In addition, various types of regulation are associated with mature females, including regulation of nucleic acid metabolism, primary metabolism, transcription, and physiological process.

GO term comparisons between males and mature females further illustrate important differences related to embryogenesis. Juvenile-enriched categories represent a subset of the female-enriched vs. male comparison (not shown). In contrast, mature-enriched GO terms include some categories not seen in other comparisons (Fig. 5E). For instance, many terms associated with purine nucleotide functions are overrepresented, such as GTPase activity and purine binding. Another set of terms involves microtubule function, such as microtubule binding and polymerization, and to transcriptional regulation (Fig. 5E).

### Conservation of sex-biased gene expression

**Figure 6:**
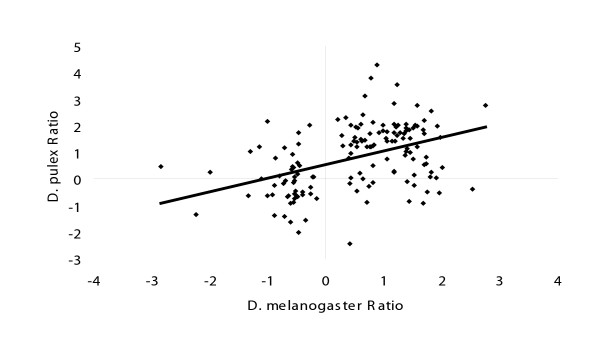
**Scatterplot of male vs. female ratios for *Daphnia *and *Drosophila***. *D. pulex *female/male ratios (females positive) are plotted on the X-axis and *D. melanogaster *female/male ratios (females positive) are plotted on the Y-axis. Genes with an e-value less than e^-08 ^were considered homologs, and all *D. pulex *genes show significant differential expression between males and females. Numbers of genes with significant homology include 27 from e^-08 ^to e^-10^; 21 from e^-11 ^to e^-20^; 54 from e^-21 ^to e^-50^; 33 from e^-51 ^to e^-80^; and 15 at e^-81 ^and lower.

At the transcriptome level, many genes show conserved patterns of expression, which can be useful for inferring gene function [40,41]. For example, many genes show sex-biased patterns of expression conserved between *D. melanogaster *and *A. gambiae *[16]. We are interested in how sex-specific gene expression patterns in *D. pulex *compare to the arthropod *D. melanogaster*. Using an expectation value (e-value) threshold of 1 × 10^-08 ^in a Blast search of our *D. pulex *sequences against the *D. melanogaster *translations (release 4.1), we retrieved 315 identifiable homologs. From this list, we found 150 genes significantly differentially expressed in our experiments, and with comparable male-female expression data in *D. melanogaster*: those of Arbeitman *et al*. [2], Parisi *et al*. [11], and Ranz *et al*. [12]. These genes display e-values with a normal distribution, and a large percentage had very strong homology. When ratio values for the *D. pulex *female/male comparison are plotted against the ratios for *D. melanogaster *(Fig. 6), several trends are apparent. Of the 150 comparisons, there are 40 differences in sign, or 73% agreement in the direction of differential expression. Linear regression shows a slope of 0.51 and an r^2 ^of 0.19, indicating a weak relationship between the magnitudes of the ratios. Genes in the comparison were analyzed for enrichment of GO terms as described above (Fig. 5F). Results show that ribosome-related categories are the most enriched, followed by those associated with protein and macromolecule metabolism, structural molecule activity, chromosome packaging, and DNA binding. For genes showing opposite trends in *D. pulex *and *D. melanogaster*, nine were metabolic, seven unknown, four proteolytic, four structural, three translational, two were signal transduction genes, and one a transporter gene.

**Figure 7:**
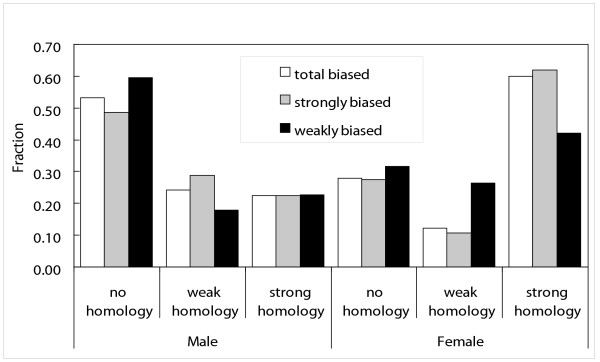
**Sequence homology and differential expression**. Histogram of sequence homology vs. count percentage for weakly biased (ratio less than 2-fold different), strongly biased (ratio greater than 2-fold different) and all biased genes in male vs. female comparison. Homology is defined as strong if bit score is 101 or greater, corresponding to an e-value of roughly 1 × 10^-20^; moderate if bit score is greater than 50, corresponding to an e-value of roughly 1 × 10^-08^; or weak if bit score is 50 or less.

Recent studies have found that the evolution of protein coding sequence is correlated with gene expression patterns [40,42,43]. A striking example is found in the rapid evolution of male-biased genes in *D. melanogaster *[12,13,42]. We asked whether *D. pulex *genes with female- or male-biased gene expression are more or less likely to be conserved than non-sex biased genes. We used a tblastx search of the GenBank NR database to assign genes into 3 categories: strong homologs (bit score > 100), weak homologs (bit score of 50–100) or no homology (bit score < 50). We further binned these genes according to their expression pattern as female-biased, male-biased, and unbiased (Fig. 7). Among these sequences, 112 female-biased genes have strong homologs compared to 89 in males. In contrast, there are 210 male-biased genes with no blast homolog but only 52 in females. Furthermore, a far greater proportion of female-biased genes have strong homologs than male-biased genes (60% vs. 23%), while a greater proportion of genes with no identifiable homolog are male-biased than female-biased (53% vs. 28%). These data indicate a greater degree of conservation of female-biased compared to male-biased genes in our set of *D. pulex *sequences.

Rapid evolution of male-biased genes may be due to either relaxation of selective pressure, or to increased positive selection favoring sequence divergence. One prediction of the former hypothesis is increased codon usage bias in slowly evolving genes [44], which could cause a spurious correlation between transcription and rate of evolution. To test this idea, we used correspondence analysis [45] to analyze codon usage statistics for male-biased, female-biased, and unbiased genes, using a conservative cutoff of four-fold difference for biased genes. We found no difference in codon usage bias between these classes as measured by frequency of optimal codons (Fop), codon bias index (CBI), codon adaptation index (CAI), or relative synonymous codon usage either at a four-fold cutoff, or at a more relaxed two-fold cutoff (data not shown). Additionally, correlations between third position GC content and Fop (r^2 ^= 0.47; p < 0.05) or CBI (r^2 ^= 0.56; p < 0.01) indicate GC content is likely to be causing major trends in codon usage among these genes [46]. These observations show that for the genes in our data set, selection on codon usage is not a cause of the relationship between protein evolution and expression bias.

## Discussion

### Gene expression analysis of males and females

Sexual reproduction is a core biological function and recent genome-wide studies of sexual dimorphism and gametogenesis in model metazoans have revealed several recurring patterns. (i) A very large proportion of the transcriptome shows some level of sex-biased gene expression [4,5,11]. (ii) The most profound sex-biased gene expression is found in the germ line [1,4,6,11]. (iii) Sex-biased genes are non-randomly distributed among sex-chromosomes and autosomes [15,47]. (iv) Male-biased genes show accelerated patterns of evolution [9,13]. Two features of *D. pulex *make it particularly interesting for a comparative study of sex-biased and reproductive genes. First, *D. pulex *are cyclically parthenogenetic, alternating sexual and asexual rounds of reproduction. Second, sex determination is environmentally induced, as males are genetically identical to their mothers (sexual reproduction results in female offspring). Thus in *D. pulex*, we expect to find the patterns of sex-biased gene expression common to the arthropods overlaid with changes associated with parthenogenetic oogenesis and environmental sex determination.

As a first step in understanding the genetic basis of the *D. pulex *reproductive system, we profiled the expression of genes differentially expressed in males compared to parthenogenetically reproducing females. *Daphnia *oogenesis proceeds with a clutch of oocytes undergoing development in the ovaries, while embryos develop in the brood chamber [26]. Shortly after molting, which releases young from the brood chamber, newly vitellogenic eggs from the ovary are deposited into the brood chamber. In the intervening intermolt period, embryonic development and vitellogenesis proceed in the brood chamber and ovary, respectively. Thus, females reproducing parthenogenetically contain both developing egg chambers as well as developing embryos. In contrast, juvenile females generally contain pre-vitellogenic oocytes but no embryos [26]. Therefore, we compared males to both juvenile females and females carrying embryos in order to partition the embryonic component from the two female types, while simultaneously ensuring a comparison with reproductively capable females.

Males used in these experiments were treated identically and showed no systematic differences in the two experiments, so the low level of two-fold differential expression in the male-juvenile female comparisons must reflect a higher level of transcript present in juvenile vs. mature females. The paucity of highly differentially expressed genes in males vs. juvenile females is likely due in part to having few male-specific transcripts on the arrays. Gene expression due to oogenesis, occurring in both juvenile and mature females, and embryogenesis, which occurs only in mature females, also shows an important contribution from embryos. Of the functional annotations of embryo-associated genes, the most prominent are related to DNA metabolism and transcription, and to microtubule function. It is interesting that in other arthropod male-female comparisons, microtubule function is male-enriched, presumably due to spermatic function [11]. It is not clear why *Daphnia *embryos are so highly enriched for this ubiquitous protein. Future studies of gene expression across the life cycle of *D. pulex *should shed light on specific embryonic genes and pathways, and provide useful insights into comparative developmental genomics of arthropods.

Patterns of sequence conservation and sex-biased gene expression in *D. pulex *are broadly similar to those seen in comparable studies of *Anopheles gambiae *and *D. melanogaster*. In particular, we found a high proportion (roughly 50%) of sex-biased expression among genes on our array, similar to other reports [3,7,12,16], with large numbers of both male- and female-biased genes. It is interesting that, in spite of potential bias toward discovering female-enriched genes on our arrays due to the nature of the arrayed cDNA library, we found a large number of genes to be male-biased. Functional categories overrepresented in males were associated with cuticle metabolism and protease function, which contrasts to the situation seen in adult mosquitoes [16]. We speculate that increased cuticle metabolism in males and juvenile females is a result of allometry (mature females being roughly double the size), although we cannot rule out the possibility of differences in cuticular composition or structure. Higher expression of proteases in males also differs from *A. gambiae*, in which female transcripts were enriched for genes associated with blood feeding, such as salivary gland proteins [16]. In mice, immune-related gene expression is also lower in testes than ovaries, although the reason for this difference is unknown [5]. Some proteases with higher expression in *D. pulex *males are homologous to digestive enzymes, but there are also clear examples of homologs of defence response proteins, as well as non-proteolytic members of these signaling pathways. It is not clear whether male *Daphnia *have a greater need for protease activity for defence, immune signaling, or metabolism. Finally, although males have higher mass-specific metabolism than females [48], females rather than males are enriched for energy metabolism-annotated GO categories.

Some of the biological processes overrepresented in female *D. pulex *are similar to those in other arthropods, such as translation elongation, ribosomal function, and DNA packaging. In comparing conserved sex-biased genes in *D. pulex *and *D. melanogaster*, we found 31 male-biased, 86 female-biased and 32 genes with opposite bias. These differences are on the same order as differences found in comparing *D. melanogaster *to *D. simulans *[12], where 20% of the genes studied had changed sex-biased expression between species. In marked contrast, Hahn and Lanzaro [16] found only 4 orthologs that had sex-biased expression in both *Anopheles *and *Drosophila*. Discrepancy between this finding and our study probably reflects the two-fold cutoff used in the *Anopheles *study [16], and difficulty in assigning orthologs to rapidly evolving male-biased genes. However, it is clear that proteins involved in translation and DNA packaging are female-biased in expression among flies, mosquitoes and *Daphnia*.

We found a difference in the degree of conservation between female and male-biased genes, similar to studies showing that female-biased genes tend to be better conserved than male-biased genes [9,14]. Data from population genetic studies indicate an increase in positive selection on male biased genes, which also have a significantly lower level of codon bias than female-biased genes. These findings were recently extended by Zhang and colleagues [13], who analyzed polymorphism levels for sex-biased and unbiased genes in several *Drosophila *species. Their results demonstrate an accelerated rate of evolution for male-biased genes, and that these genes are more often subject to positive selection [13]. It is clear that for genes in our data set, selection on codon usage is not driving differences in evolutionary rates, nor is it causing a spurious correlation between bias and rate. However, it is possible that our data set was too small to detect codon usage differences.

Future studies should focus on examining differences in population polymorphism and species divergence, for example in the nonsynonymous/synonymous polymorphism ratio in biased genes between populations of *D. pulex *and *D. magna*, a congener for which cDNA data are accumulating rapidly. This comparison would distinguish between relaxed selective constraints or increased positive selection on male genes. The ability to control for chromosomal effects makes a useful comparison to chromosomally-determined systems, in distinguishing whether linkage or some other mechanism is responsible. Another central question is whether male-biased genes are mutationally degenerating in non-male producing obligate asexual lines, and we identify candidate genes for such research. Finally, comparing parthenogenetic reproduction to sexual reproduction in this species should also yield interesting candidates for molecular evolutionary studies, and provide clues as to the mechanisms involved in these processes. The public release of the assembled *D. pulex *genome, combined with genetic mapping studies [49], should provide insight into the roles of recombination rate, chromosomal context, demographic history, and selection on shaping sequence divergence in sex-biased genes.

## Conclusion

In this study, we analyze patterns of gene expression differences in *D. pulex *males, juvenile females, and pregnant females. We find patterns of sex-specific and pregnancy-specific expression across a substantial proportion of genes. Results suggest that patterns of female-biased expression are similar to those reported for *D. melanogaster *and *A. gambiae*, indicating conserved functional requirements across arthropods. Specifically, females express transcripts relating to protein metabolism, primary metabolism, and organelle function at a higher level than do males. Embryonic gene expression is enriched for processes related to DNA metabolism, transcription, and microtubule function. Male-biased transcription is over-represented with cuticle and protease functions, in contrast to other arthropods. We also find that male-biased genes are less likely to have identifiable homologs in sequence databases, consistent with faster evolution of male-biased genes.

## Methods

### Microarray Construction

We constructed the first *D. pulex *microarray. The arrays were printed with 3,602 PCR amplified cDNA clone inserts and 240 positive and negative controls, corresponding to 787 clusters of nuclear-derived genes as described [30]. cDNA libraries were constructed as described elsewhere [30]. The printed array elements were from a mixture of anonymous clones and clones with single pass 5' EST sequence reads. Fabrication of microarrays was conducted concurrently with EST sequencing, and we have sequenced 1,546 high-quality clones on the array to date. A detailed description of this platform has been submitted to the Gene Expression Omnibus at NCBI under the accession number GPL4349, series GSE5908. Not all of the arrayed clones were sequenced, and many genes were represented by multiple clones. Therefore, we define redundancy on the array as the proportion of unique sequences among our clones. Given approximately 57% redundancy among the sequenced clones [30], we estimate that the array contains 1,560 distinct *D. pulex *genes. From each library, 384 samples were PCR amplified from plasmid purified using the PerfectPrep kit (Eppendorf) and sequenced using BigDye (ABI; v.2) on an ABI 3730 sequencer. These samples served as a random sample for quality control in further analysis. Remaining samples (3,168 in all) were PCR amplified from colony picks of transformed bacterial cells. Template was generated by growing colonies in 1.2 ml of 2× YT and 0.005% chloramphenicol in 96-deepwell plates for 24 hours at 37°C. Reactions were run in 100 μl containing: 1× Taq buffer (Eppendorf), 0.2 mM dNTPs, 0.2 μM primers (Fwd. 5'-GTGTAAAACGACGGCCAGTAG 3' and Rev. 5'-AAACAGCTATGACCATGTTCAC 3'), 5 U Taq (Eppendorf), and 5 μl fresh bacterial growth or 2 μl purified plasmid. Quality of PCR amplifications was verified by gel electrophoresis and number and size of bands was recorded using Kodak's 1D imaging software (v.3.6). Concentration of samples was determined by 96-well microplate spectrophotometer (Molecular Devices SpectraMax 190) and adjusted to 50–200 ng/μl for printing.

A Genemachines Omnigrid 100 was used to print the *D. pulex *cDNA on GapsII aminosilane slides (Corning). The cDNA was printed in 3× SSC and 1.5 M Betaine buffer and deposited with Stealth Micro-Spotting Pins (Telechem) at 20°C and 65% humidity. The cDNA was fixed to the microarray slides by baking at 85°C for 3 hours. Slides were processed by agitating in 0.2 % SDS for 5 min. and in HPLC-grade water, 5 min at 21°C., followed by 2 min. in 95°C water, rinsing briefly in ice-cold 100% isopropanol, and centrifuging at 500 × *g *for 5 min. Five types of negative controls were printed, including printing buffer, failed PCR reaction with template DNA but no primers, ORFs from *Arabidopsis *and lambda phage, and bacterial PCR spikes (Ambion). Positive controls of *D. pulex *genes were printed next to printing buffer to assure no carryover, and included cytochrome *c *(subunits I, II, and III), cytochrome b, actin, and ferritin.

### Animal Culturing and Experimental Design

*Daphnia *were reared in lake water at 20°C and a 10:14 light/dark cycle at a density of approximately 1 individual per 5 ml. Animals were fed *Scenedesmus *algae at approximately 0.1 mg ml^-1 ^each day. Twenty animals of a single clone were exposed to 400 nM methyl farnesoate in methanol (60 μl L^-1^) to induce male production, while another 20 individuals were given a sham control of methanol and produced female offspring. Progeny were raised under conditions described above in eight beakers each for males and females, with about 25 individuals per beaker and inspected by microscopy to verify healthy appearance and correct sex. After six days, beakers containing juvenile females were sacrificed. After eight days, males and the remaining females (now carrying embryos in their brood pouches) were harvested as well. Total RNA was isolated using Trizol (Invitrogen) and RNeasy columns (Qiagen), including a DNase treatment performed on-column. Quality of total RNA preparations was assayed by spectrophotometry and electrophoresis through denaturing agarose gels.

Four biological replicates of juvenile females and adult females were compared to eight biological replicates of males. Two samples of each female replicate were labeled with red and two with green dye, and no technical replicates were performed. Eight total hybridizations were therefore performed.

### Microarray Hybridization, Analysis, and Validation

Isolated total RNA (15 μg) was reverse-transcribed into first-strand cDNA using the SuperScriptIII indirect labeling kit (Invitrogen), and coupled to Alexa dyes 555 and 647 (Invitrogen). Incorporation was assessed by spectrophotometry and gel electrophoresis followed by image quantitation on a Typhoon phosphorimager (Molecular Dynamics). Results showed good incorporation of fluor, a size range of cDNA into the 1 kb range, and negligible amounts of unincorporated dye. Equal masses of cDNA from males and females were combined in a 1:3 volume with hybridization buffer (50% formamide, 5× SSC, 0.1% SDS, 10 μg calf thymus DNA) and denatured at 90°C for 4 min, quick chilled, and injected into a Lucidea Slidepro automated hybridization chamber (Amersham) containing a microarray slide pre-hybridized according to manufacturer's instructions. Following overnight hybridization, slides were washed twice at 60°C in 2× SSC + 0.2% SDS (15 min), once in 0.2× SSC + 0.2% SDS (10 min), once in 0.1× SSC (10 min) and once in 0.05× SSC (10 min). Slides were dipped briefly in 100% isopropanol, centrifuged 5 min at 500 × *g *to dry, and scanned using an Axon 4200B scanner.

Data were extracted using GenePix software (version 5.1) and imported into Bioconductor for normalization and analysis [50]. Data were compared using multiple methods, including several Bioconductor packages. Data were normalized using OLIN [51] or limma [33] and differential expression was assessed with limma and EBarrays [52]. Array elements are defined as differentially expressed in our analyses if they have a p-value less than 0.05 with a false discovery rate correction using the program linear models for microarrays (Limma) [33] implemented in Bioconductor [50].

We compared analyses using OLIN for normalization and EBarrays for differential expression, or using limma for both. Briefly, we examined assumptions of the analytical models using constant coefficient of variation and quartile-quartile plots, which showed reasonably good fits. However, certain contrasts in EBarrays were not as well fit as others for our experimental dataset, so we used the linear model framework. We performed normalizations using print-tip loess normalization with no background subtraction after genes < 2 S.D. above median background were filtered out. Gene ontology category assignment and analysis was performed using the program Blast2GO [39], with input data consisting of assembled sequences called as differentially expressed by Limma using a 5% false-discovery rate. Codon statistics were generated using correspondence analysis [46] as implemented in the program codonW [45]. For these analyses, significantly differentially expressed genes were binned as biased if the male-female difference in both juvenile and mature comparisons was greater than 4-fold (arithmetic scale).

For clarity, we describe gene expression patterns in terms of (i) the number of array elements (to distinguish from spots, which are printed in duplicate), (ii) the number of sequenced, non-redundant genes (which we also call "assembled" sequences) and (iii) the estimated number of unique genes represented by those elements.

Quantitative PCR assays were performed with the SuperScript III Real-time PCR kit (Invitrogen) following manufacturer's protocol for 2-step amplification, using BioRad's I-cycler. Briefly, DNase-treated total RNA (200 ng) was reverse transcribed in 20 μl and amplified in duplicate samples from two separate RNA preparations. Reactions consisted of 200 μM dNTPs, 3 μM gene-specific primers, 1 μM fluorescein, and 2 U Taq. A standard curve was constructed using cDNA as template, and reactions were subjected to melt-curve analysis after amplification, which revealed a single band in each case. Reaction products were also subjected to agarose gel electrophoresis in some cases to verify amplification of a single species. Sequences of primers used were as follows: 01031D01 F 5'CGCTTCTTCTGCCTATCTGC 3';

01031D01R 5' GAAGAAAGCTGCGAATGTCC 3'; 02081H07F 5'

CGGAAATCCTTCCCACTACA 3'; 02081H07R 5'

GGGAGCGTAGTTGTCACCAT 3'; 01001A03F 5'

TTACCCATCTGCCGTCTACC 3'; 01001A03R 5'

GATTTAAACGGCAGCGAATC 3'; 02081G03F 5'

TCCACTGACATTGGCGTTTA 3'; 02081G03R 5'

CCAAATCATTGGCAAATTCC 3'; 02102C08F 5'

TCACCAAATTCGTTCCAACA 3';

02102C08R 5' TCGGGCTTCATGTTATCTCC 3'.

## Authors' contributions

BE carried out microarray studies, sequence collection and data analysis; JC performed data and sequence analysis; EB characterized the cDNA libraries, created the microarrays and assisted in quality control and sequence collection; JA assisted in coordinating and designing the study and drafting the manuscript. All authors read and approved the manuscript.

## Supplementary Material

Additional file 1 File is a tab-delimited text file containing microarray expression data for four different experimental comparisons, including ratio, intensity and p-values for all elements on the array, as well as homology data about the elements. Click here for file
